# The clinical application of array CGH for the detection of chromosomal defects in 20,126 unselected newborns

**DOI:** 10.1186/1755-8166-6-21

**Published:** 2013-06-01

**Authors:** Sang-Jin Park, Eun Hye Jung, Ran-Suk Ryu, Hyun Woong Kang, He Doo Chung, Ho-Young Kang

**Affiliations:** 1 MG MED, Inc., 60-24, Gasan-dong, Seoul, Korea; 2 MACROGEN, Inc., Seoul, Korea; 3 MGMED Clinic, Seoul, Korea

**Keywords:** Array CGH, Newborns, Chromosome abnormality

## Abstract

**Background:**

Array comparative genomic hybridization (CGH) is a powerful tool for detecting unbalanced chromosomal alterations. To validate the usefulness of array CGH in newborn screening, we examined 20,126 unselected infants. In addition, the number of newborns analyzed with array CGH is the largest one ever reported.

**Findings:**

A total of 20,126 unselected newborns were investigated with array CGH and cytogenetic analyses. The analyses revealed 87 cases with chromosome abnormalities. Of these, 53 cases had significant chromosome aneuploidies, including trisomy 13, trisomy 21, 47,XXY or 45,X, and the other 34 cases presented partial chromosomal deletions or duplications.

**Conclusions:**

In this study, we show that array CGH is an appropriate tool for the screening of chromosomal abnormalities in newborns, especially for the infants without distinct clinical features.

## Background

Array Comparative Genomic Hybridization (CGH) was developed as a screening strategy for detecting genome-wide DNA copy number changes and many groups have studied the clinical applications of array CGH in both prenatal and postnatal settings [1-5]. Chromosomal abnormalities are a major cause of congenital and developmental abnormalities in human genetic diseases, associated with dysmorphic features, mental retardation and developmental delays, as well as multiple congenital anomalies. The most common chromosome abnormalities in newborns are trisomy 21 and sex chromosome abnormalities. The early diagnosis of these chromosomal disorders is very important to achieve optimal management and treatment [6,7]. The International Standard Cytogenomic Array (ISCA) Consortium published a consensus statement on the use of chromosomal microarray as a first tier diagnostic test in the evaluation of individuals with developmental delays and/or congenital anomalies [4]. Our group successfully developed and validated a bacterial artificial chromosome (BAC)-based array CGH analysis platform including analysis software [8].

In this study, we investigated 20,126 unselected newborns with array CGH and identified 87 abnormal cases.

## Results

**Figure 1:**
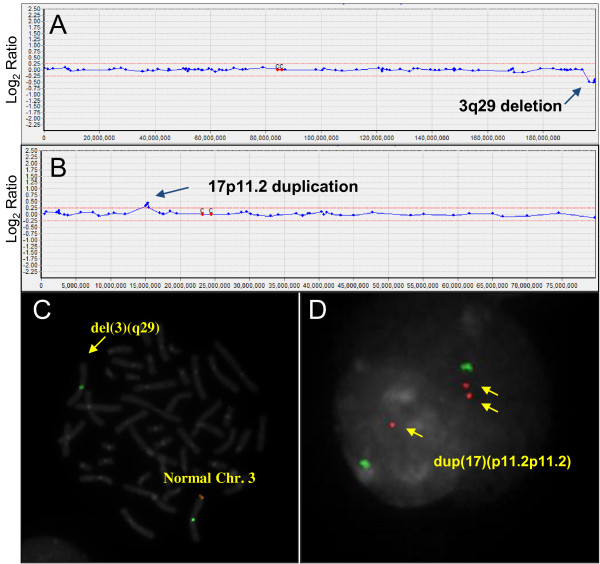
**Examples of Array CGH and FISH results for 3q29 microdeletion case (A, C) and 17p11.2 duplication case (B, D).** (**A**) The array CGH result for chromosome 3. Arrow indicates deletion of the 3q29 region including the *PAK2* and *DLG1* genes. (**B**) The array CGH result for chromosome 17. Arrow indicates duplication of the CMT1A region (17p11.2). (**C**) FISH result analyzed with a 3q29 region specific probe; arrow indicates a deletion of 3q29 region in chromosome 3. (**D**) FISH with 17p11.2 region specific probe; arrows indicate a duplication of 17p11.2 region in an interphase cell.

Whole-genome array CGH analysis was performed as a first line test to screen for genomic imbalances in 20,126 unselected newborn infants (Table 1). We simultaneously performed FISH and a G-banding analysis to confirm abnormal results of array CGH data. Of 20,126 neonatal cases, 0.43% (87/20,126) had DNA copy number variations: 53 cases of aneuploidy, 23 deletions, and 11 duplications (Table 2). Among the 53 aneuploidies, 18 cases were autosomal aneuploidies (18/20,126; 0.08%), and 35 cases involved sex chromosomes (35/20,126; 0.17%). The most frequent chromosomal abnormality was trisomy 21, and 47,XXY was the next most common aneuploidy. Gains or losses associated with chromosomal deletion (microdeletion) or duplication (microduplication) were observed in 34 cases. For examples, 3q29 microdeletion syndrome (arr[hg19] 3q29(196,412,227-197,102,739) × 1), Cri-du-chat syndrome (arr[hg19] 5p15.2p15.3(9,458,494-9,803,306) × 1), Soto’s syndrome (arr[hg19] 5q35.2q35.3(176,464,673-176,795,643) × 1), Prader-Willi/Angelman syndrome (arr[hg19] 15q11.2(24,055,918-27,026,553) × 1), DiGeorge syndrome (arr[hg19] 22q11.2(19,030,620-19,861,970) × 1), Steroid sulfatase deficiency (arr[hg19] Xp22.31(7,078,532-7,676,445) × 1), Hereditary neuropathy with liability to pressure palsies (arr[hg19] 17p11.2(15,067,223-15,225,580) × 1), and Charcot-Marie-Tooth Disease type 1 (arr[hg19] 17p11.2(15,067,223-15,225,580) × 3). We also identified 15q11.2q12 interstitial duplication which is validated by karyotyping and FISH as 46,XY.ish dup(15)(q11.2q12)(SNRPN+). And Inverted duplication 15q case involving mosaicism and a small supernumerary marker chromosome (sSMC) were also found. We identified various microdeletion or duplication cases and the molecular cytogenetic results are shown in Figure 1.

**Table 1:** Summary of array CGH analysis

	**Cases with abnormal array CGH analysis**^**a**^			**Total (N)**	**Detection rate (%)**
	**Aneuploidy (N)**	**Deletion (N)**	**Duplication (N)**		
Neonatal cases^b^					
(N = 20126)	53	23	11	87	0.43

^a^ array CGH analysis results confirmed by Karyotyping and FISH analyses.

^b^All neonatal cases, collected < 0.5 year of age, between 2010–2012.

**Table 2:** Summary of abnormal cases

**Array CGH analysis**	**Cytogenetic analyses **^**a**^	**Disorder**	**No. cases**
	**Aneuploidy**		
Duplication of whole chr.13	Trisomy 13	Patau syndrome	1
Duplication of whole chr.21	Trisomy 21	Down syndrome	17
Duplication of whole chr.X	47,XXY	Klinefelter syndrome	15
Duplication of whole chr.Y	47,XYY	XYY	9
Duplication of whole chr.X	47,XXX	Triple X	8
Deletion of whole chr.X	45,X	Turner syndrome	2
Deletion of whole chr.X	mos 45,X[28]/ 46,X,i(X)(p22.3 → q22::q22 → p22.3)[2]	Turner syndrome	1
	**Deletion/ Microdeletion**		
Deletion of 0.6 Mb at 3q29	46,XY.ish del(3)(q29)(PAK2-)	3q29 microdeletion	1
Deletion of 0.3 Mb at 5p15.2p15.3	46,XX.ish del(5)(p15.2p15.3)(D5S727-)	Cat cry syndrome	2
Deletion of 0.3 Mb at 5q35.2q35.3	46,XY.ish del(5)(q35.2q35.3)(NSD1-)	Sotos syndrome	1
Deletion of 3 Mb at 15q11.2	46,XX.ish del(15)(q11.2q11.2)(SNRPN-)	Prader-willi/Angelman syndrome	1
Deletion of 0.3 Mb at 17p11.2	46,XY.ish del(17)(p11.2p11.2)(PMP22-)	HNPP	2
Deletion of 0.3 Mb at 17p11.2	46,XX.ish del(17)(p11.2p11.2)(PMP22-)	HNPP	4
Deletion of 0.8 Mb at 22q11.2	46,XY.ish del(22)(q11.2q11.2)(TBX1-)	DiGeorge syndrome	2
Deletion of 0.6 Mb at Xp22.31	46,XY.ish del(X)(p22.31p22.31)(STS-)	Steroid sulfatase deficiency	7
Deletion of 53 Mb at Xp11.2pter/	46,X,i(X)(q10)	Sex chromosome abnormality	1
Duplication of 94 Mb at Xq10qter			
Deletion of 55 Mb at Xq21qter	46,X,del(X)(q21qter)	Sex chromosome abnormality	2
	**Duplication/ Microduplication**		
Duplication of 0.3 Mb at15q11.2	46,XY.ish dup(15)(q11.2q11.2)(SNRPN+)	15q duplication	1
Duplication of 0.2 Mb at17p11.2	46,XY.ish dup(17)(p11.2p11.2)(PMP22+)	CMT1A	2
Duplication of 0.8 Mb at22q11.2	46,XY.ish dup(22)(q11.2q11.2)(COMT+)	22q11.2 duplication	5
Duplication of 0.8 Mb at22q11.2	46,XX.ish dup(22)(q11.2q11.2)(COMT+)	22q11.2 duplication	2
	**Small supernumerary marker chromosome**		
Duplication of 2 Mb at 15q11.2q13	mos 47,XX,+inv dup(15)(q11.2q13)[9]/ 46,XX[11]	Inverted duplication 15q	1

HNPP, Hereditary neuropathy with liability to pressure palsies; CMT1A, Charcot-Marie-Tooth Disease type 1.

^a^ Karyotyping and FISH analyses.

## Discussion

Microarray-based comparative genomic hybridization (array CGH) is a high-resolution and comprehensive method for detecting both genome-wide and chromosome-specific copy-number imbalance. We have developed an array CGH analysis system for constitutional genetic diagnosis and have evaluated the suitability of our system for molecular diagnosis. Our array CGH chip consists of 1,440 non-overlapping bacterial artificial chromosome (BAC) clones, which were selected from 96,768 BAC clones constructed by the Korean Genome Project and validated by end-sequencing and FISH [8,9]. Therefore, the abnormal array CGH results were able to be confirmed by FISH.

Several studies of unbalanced chromosomal abnormalities in newborns have reported prevalence rates of 17 ~ 31/10,000 live births [10-12]. Recently, Wellesley *et al*. reported that the overall frequency of unbalanced chromosome abnormalities was 0.43% (43.8/10,000) [7]. Of these, 0.36% was significant chromosome aneuploidies (T21, T18, T13, sex chromosome trisomies, and 45,X) and 0.07% was rare chromosome abnormalities (triploidy, other trisomies, marker chromosomes, unbalanced translocations, deletions, and duplications). Similarly, we observed abnormalities in 0.43% of 20,126 unselected newborn infants (Table 1). Of these, 0.26% (53/20,126) was characterized as significant chromosome aneuploidy. Regarding rare chromosome abnormalities, we found 0.17% of frequency which is much higher than 0.07% reported by Wellesley *et al*. The difference is due to different methods to detect chromosome abnormalities.

Trisomy 21 and sex chromosome aneuploidy (XXY, XXX, XYY and 45,X) were the most frequent abnormalities (Table 2). In addition to the high frequency of diseases associated with aneuploidies, detecting chromosomal abnormalities at an early age is very important for the optimal management and treatment of the affected newborns. For example, patients with Turner syndrome (TS) can be treated with growth hormones if they are diagnosed early in life. However, many girls with TS are not diagnosed until after 10 years of age, thus resulting in delayed evaluation and treatment [13]. Although Klinefelter syndrome is mainly diagnosed in pre-pubertal males, early identification and anticipatory guidance are extremely helpful [14]. In Down’s syndrome, early identification makes it easier to achieve the goals of treatment, particularly controlling the symptoms and managing the resulting medical conditions [15].

The chromosome deletions and duplications identified in the remaining 34 abnormal cases are associated with many clinical indications, such as developmental delays and mental retardation. The 22q11.2 deletion syndrome, also known as DiGeorge syndrome or velocardiofacial syndrome, is a genetic disorder with multisystemic manifestations, including congenital cardiac abnormalities, palatal anomalies, T-cell immunodeficiency, craniofacial features, cognitive deficits and schizophrenia [16,17]. The early diagnosis of and early intervention for psychiatric illnesses improve the long-term prognosis in individuals with schizophrenia and bipolar disorder [18]. Other treatments can usually correct critical problems, such as heart defects or low calcium levels [19].

The 5p15 deletion syndrome, known as “cat cry” or Cri du Chat syndrome, has clinical features such as low weight, microcephaly, round face, large nasal bridge, hypertelorism, epicanthal folds, downward-slanting palpebral fissures, down-turned corners of the mouth, abnormal dermatoglyphics, and a characteristic sounding cry in infancy [20]. We also identified 15q11.2 duplications. The symptoms associated with these duplications appear to range from minor (apparently normal) to highly severe mental retardation, growth retardation, and autism [21]. The early recognition of Charcot-Marie-Tooth type 1, which is caused by a 17p11.2 duplication, can prevent life-threatening vincristine neurotoxicity [22].

Chromosomal disorders with developmental delays or mental retardation may not be recognized until a certain developmental stage. Some patients who have chromosomal aberrations, such as 47,XXY or 45,X, do not exhibit clinical features until after a certain year of age. Although there are no cures for chromosomal disorders, many patients without distinct clinical features can be effectively managed and treated in the early stages of development by early diagnosis with array CGH.

In this study, we showed that our newly developed array CGH platform is very useful for clinical application in newborns, especially for the infants without distinct clinical features. In addition, the number of newborns analyzed with array CGH is the largest one ever reported.

## Materials and methods

### Patient samples

We analyzed samples obtained from 20,126 unselected neonates who had been referred to MGMED laboratories for array CGH analysis between January 2010 and December 2012. A total of 20,126 unselected neonatal samples (16,850 peripheral blood and 3,276 cord blood) were collected for chromosome abnormality screening. All samples were prepared for experiments using previously described methods [8]. All patient materials were obtained and evaluated with informed patient consent and with the approval of the Ethics Committees of the MGMED clinical center.

### Array CGH and cytogenetic analyses

Approximately 100 ~ 200 ng of DNA was used for the array CGH experiments, as described, with slight modifications [2]. The slides contained 1440 human BAC clones including specific loci for more than 50 chromosomal disorders. Briefly, DNA was labeled with Cy-3 and Cy-5 dCTP by a random priming method for 3 h. The labeled DNA was purified, dissolved in hybridization buffer and hybridized overnight. The slides were washed several times and dried. Slide images were acquired with a GenePix4000B dual-laser scanner (Axon Instruments, Union City, CA) and analyzed with MacViewer software. Chromosome analysis was performed according to standard methods using cultured cells from peripheral blood samples obtained from the patient. FISH studies on interphase or metaphase spreads with specific probes were performed as described [8]. Cytogenetic analyses were described according to the conventions of the International System for Human Cytogenetic Nomenclature (ISCN, 2013).

## Competing interests

The authors declare that they have no competing interests.

## Authors’ contributions

SJP drafted the manuscript and analyzed the data for the paper. HDC helped with the discussion and data summary. EHJ, RSR and HWK performed various experiments. HYK conceived of the study and approved the final manuscript. All authors read and approved the final manuscript.
